# COVID vaccine evaluation of barriers and resources among families of children with diagnosed allergies

**DOI:** 10.3389/falgy.2023.1101247

**Published:** 2023-05-05

**Authors:** Gregory D. Gooding, Jennifer L. Protudjer, Sofianne Gabrielli, Pasquale Mulé, Greg Shand, Xun Zhang, Christine McCusker, Francisco J. Noya, Maria Harvey, Mélodie Chalifour, Catherine Sicard, Elissa Abrams, Jacques-Alexandre Amiel, Thanh-Thao Ngo, Andre Bonnici, Noni MacDonald, Moshe Ben-Shoshan

**Affiliations:** ^1^ Division of Allergy and Clinical Immunology, Department of Pediatrics, Montreal Children’s Hospital, McGill University Health Centre, Montreal, QC, Canada; ^2^ Department of Pediatrics and Child Health, University of Manitoba, Winnipeg, MB, Canada; ^3^ The Children’s Hospital Research Institute of Manitoba, Winnipeg, MB, Canada; ^4^ Institute of Environmental Medicine, Karolinska Institutet, Stockholm, Sweden; ^5^ Department of Food and Human Nutritional Sciences, Faculty of Agricultural and Food Sciences, University of Manitoba, Winnipeg, MB, Canada; ^6^ George and Fay Yee Centre for Healthcare Innovation, Winnipeg, MB, Canada; ^7^ Department of Pharmacy, Montreal Children’s Hospital, McGill University Health Centre, Montreal, QC, Canada; ^8^ Department of Paediatrics, Dalhousie University, IWK Health Centre, Halifax, NS, Canada

**Keywords:** allergy, children, family, COVID-19, vaccine hesitancy, knowledge translation

## Abstract

**Background:**

We aimed to determine vaccine hesitancy and the main barriers associated with the 2019 novel coronavirus, SARS-CoV-2 (COVID-19) vaccination among families of children diagnosed with food/drug/environmental allergies.

**Methods:**

Between May and June 2021, we approached 146 families seen at the outpatient allergy clinic at the Montreal Children's Hospital and a community allergy practice were invited to complete an anonymous online survey on COVID-19 and vaccination attitudes and behaviour. Uni and multivariable logistic regressions were compared to estimate factors associated with vaccine hesitancy.

**Results:**

Among all patients, 24.1% reported vaccine hesitancy. The large majority of parents (95.2%) believed that vaccines work. The most common barrier to vaccination was fear of adverse side effects (57.0%). One-third of participants (31.5%) reported that a history of food, venom and drug allergy was a contraindication for COVID-19 vaccination. Fifty-nine (60.8%) participants stated that the dissemination of additional information would increase their willingness to be vaccinated. Most (96.9%) parents reported that their children's vaccinations were up to date. Hesitant families were more likely to be parents of children aged 6–10 years, be of Asian descent, report that mRNA vaccines are riskier than traditional vaccines, and report that the vaccine should not be given if the child has a history of allergic reaction to vaccines.

**Conclusion:**

Vaccine hesitancy exists mainly among certain ethnic groups and families with young children. Allergies to food, venom and drug allergy are commonly perceived as contraindications for COVID-19 vaccination. Knowledge translation activities addressing parental concerns will help increase vaccination rates.

## Introduction

The implementation of COVID-19 vaccinations is suggested to play a crucial role in pandemic control. Yet, COVID-19 vaccination rates remain variable and suboptimal globally, ranging from <5% to 99% (1).

In children, vaccines may reduce the risk for developing severe disease manifestations, such as multisystem inflammation syndrome (MIS-C), high susceptibility to persistent COVID-19, and transmission of certain variants (2–4).

It is especially important to assess hesitancy among children with known food/drugs/aeroallergen allergies given that vaccination was listed as a contraindication for patients with food or drugs allergy and as a precaution in patients with aeroallergy according to recommendations by the Medicines and Healthcare Products Regulatory Agency (MHPRA) regulates medicines (5). We aimed to determine vaccine hesitancy and the main barriers associated with COVID-19 vaccination among families of children diagnosed with food, venom or drug allergies.

## Methods

Parents of children at the allergy clinic at the Montreal Children's Hospital and a community allergy practice, in Montreal, Canada, were invited, in person, to complete a survey, available in either French or English, on COVID-19 and vaccination attitudes and behaviour. The questionnaire was developed by a multidisciplinary team composed of nurses, physicians, pharmacists and researchers. Parents were queried on age, sex of both parent and child, ethnicity, education level, history of vaccination of their child, parental willingness to vaccinate their child, and potential barriers for vaccination. The questionnaire was developed in English, then validated among 8 families and translated per World Health Organization guidelines for the translation of instruments, prior to distribution (6). Families provided informed consent and assent (in children at least 7 years old). Data were analysed with R (version 4.0.0). Descriptive statistics were used for reporting demographic factors (age, sex, ethnicity, education level). Uni - and multivariable logistic regressions were compared to estimate factors associated with vaccine hesitancy. Ethics approval for this study was granted by the McGill University Health Centre Research Ethics Board.

## Results

Between May and June 2021, 146 families were approached and all consented to participate in our anonymous survey (Supplementary Table 1). The largest group (26%) of children were 6–10 years old (range: 0–18 + years). The majority of parents (55.2%) were 40–49 years old (range: 20–60 + years) and had at least college education (83.6%). Among participants 110 (75.3%) indicated they would vaccinate their children for COVID-19 (95%CI: 67.9%, 82.4%). The large majority of parents 139 (95.2%) believed that vaccines work.

Among all patients, thirty-five (24.1%) reported vaccine hesitancy. Fifty-six parents (38.4%) reported hesitancy regarding vaccinating a child that had already been diagnosed with COVID-19 and over half (61.6%) had concerns regarding vaccination in pregnant women. Eighty-five (58.2%) reported hesitancy regarding vaccine safety for lactating women and/or their children. One-third of parents (34.9%) were hesitant about the safety of mRNA vaccines compared to other types of vaccines. Compared to non-hesitant families, vaccine-hesitant families were more likely to be parents of children aged 6–10 years (OR: 2.24, 95% CI: 1.04, 4.86), be of Asian descent (OR: 4.52, 95% CI: 1.56, 13.10), report that mRNA vaccines are riskier (OR: 2.43, 95% CI: 1.05, 5.61), and report a history of allergic reaction to vaccines as a contraindication (OR: 4.65, 95%CI: 1.51, 14.31) (Table 1).

**Table 1:** Univariable and multivariable analyses (OR, 95%CI) of factors associated with vaccine hesitancy.

Factor	Unadjusted	Adjusted*
Education (Post-Secondary or high school = 0, Higher than Post-Secondary = 1)	1.32 (0.41, 4.24)	1.24 (0.37, 4.41)
Trusted source		
Nurse	0.37 (0.17, 0.80)	0.37 (0.16, 0.85)
Pharmacist	0.22 (0.10, 0.50)	0.24 (0.10, 0.56)
Traditional news	0.38 (0.17, 0.86)	0.39 (0.16, 0.91)
Believe that vaccine works	0.11 (0.02, 0.60)	0.13 (0.02, 0.78)
Child age (years)		
0-2	0.85 (0.33, 2.18)	0.71 (0.26, 1.98)
3-5	1.12 (0.49, 2.55)	1.23 (0.54, 3.00)
6-10	2.24 (1.04, 4.86)	2.64 (1.16, 6.00)
15-17	0.36 (0.13, 0.99)	0.38 (0.13, 1.08)
Ethnic group (Asian descent)	3.89 (1.40, 10.77)	4.52 (1.56, 13.10)
mRNA vaccine hesitancy	2.58 (1.18, 5.63)	2.43 (1.05, 5.61)
Maternal and child health		
Vaccine safe for Women and Fetus	0.26 (0.10, 0.67)	0.24 (0.09, 0.65)
Vaccine safe in Lactating Women and Child	0.17 (0.06, 0.46)	0.18 (0.06, 0.52)
Conditions in which vaccine should not be given (Allergies, Pre-existing condition, COVID+)	3.03 (1.16, 7.91)	4.65 (1.51, 14.31)
Children vaccinated before the age of 6	1.62 (0.18, 14.35)	1.56 (0.16, 14.73)
Vaccinate previously diagnosed COVID	0.10 (0.04, 0.25)	0.07 (0.03, 0.21)
COVID vaccine info with flu vaccine	0.45 (0.21, 0.99)	0.48 (0.21, 1.10)
Child complete immunization before the age of 2	0.63 (0.06, 7.16)	0.74 (0.06, 9.40)
Willingness to vaccinate with lay explanation	0.29 (0.13, 0.64)	0.32 (0.14, 0.74)

^*^ Adjusted for age of parents, sex of parents, age of children and ethnicity.

Fear of side effects was identified as the most common primary barrier to vaccination (57.0%). The majority of parents (60.3%) stated that receiving information regarding the safety of administration for the COVID-19 vaccine at the same time as other vaccines such as the seasonal influenza injection would increase their likelihood to get vaccinated.

## Discussion

To our knowledge, we conducted the first Canadian study to assess COVID-19 vaccine hesitancy among families. Our findings reveal that 24% of parents are hesitant regarding child vaccination. Parents of younger children and parents of Asian descent were more likely to be hesitant.

Our findings have particular importance given the ever-changing landscape of the pandemic. In October 2021 children aged 10–19 years accounted for almost 15% of all newly reported cases of COVID-19 (4). It is important to note that morbidity and mortality are reported to be particularly increased among adolescents with underlying illnesses—such as diabetes, cancer, HIV, and obesity (4). The majority of families of children with food, drug or, venom allergies reported that they would appreciate lay educational tools addressing their concerns, mainly the risk of side effects. Indeed, numerous studies suggest that some of the most effective interventions that succeed in reducing vaccination hesitancy are those that aim to increase vaccine knowledge (7).

Over half of our sample ranked fear of side effects as the most important barrier to vaccination despite the fact that data reveal that benefits associated with COVID-19 vaccination and booster shots largely outweigh the risks (8). Current literature states that mRNA vaccines are safe and effective for the vast majority of the population (8). Of note, at the time of our study, regulatory bodies were not yet investigating the safety of vaccines in children aged 0–5 years which could explain why parents of these age groups were hesitant. Of particular interest, one-third of participants indicated a history of food, drug, venom, or environmental allergy as contraindications to vaccination. In Canada, the National Advisory Council on Immunizations (NACI) and the Canadian Society of Allergy and Immunology (CSACI) has stated that the vaccine is contraindicated only in persons with a history of anaphylaxis to the COVID-19 vaccine or a vaccine excipient (9).

A limitation is that the small validation group and that families recruited at the allergy clinic are more likely to be concerned regarding side effects it is possible that we overestimated vaccine hesitancy. Interestingly, a study among French-speaking health care workers in France, parts of Belgium and Quebec revealed a similar percentage of vaccine hesitancy (10).

Vaccine hesitancy is not negligible; our data suggests that it exists mainly among certain ethnic groups and for young children. Despite current evidence, allergies to food, venom and drug allergy are often perceived contraindications for COVID-19 vaccination. Knowledge translation activities such as educational videos will increase vaccination rates among children and contribute to better control of the COVID-19 pandemic.

## Data Availability

The original contributions presented in the study are included in the article/**Supplementary Material**, further inquiries can be directed to the corresponding author.
