# Supplementary material for: COVID vaccine evaluation of barriers and resources among families of children with diagnosed allergies
Source: Front Allergy. 2023 May 5;4:1101247. doi: 10.3389/falgy.2023.1101247 (PMC10198258; doi:10.3389/falgy.2023.1101247)
Supplement: Supplementary file 1 [file Table1.pdf]

Supplemental Table:

Variable		
Highest level of completed education	High school	6
	Post-secondary or equivalent	14
	College or bachelor's degree university	57
	University in a health sciences field	16
	Professional or graduate degree	42
	Other	11
Age of children	0-2	33
	3-5	44
	6-10	61
	11-14	57
	15-17	40
	18+	14
Age of parents	20-39	50
	40-49	80
	50-60+	15
	Prefer not to answer	1
Sex of parents	Male	32
	Female	113
	Prefer not to answer	1
Ethnicity	North American /Indigenous	40
	European origins	73
	Caribbean, Latin, Central and South American origins	8
	African origins	6
	Asian origins	19
	Oceania origins	0
